# Evaluation of Force Degradation Pattern of Elastomeric Ligatures and Elastomeric Separators in Active Tieback State

**DOI:** 10.15171/joddd.2015.045

**Published:** 2015-12-30

**Authors:** Amir Mohammadi, Farhang Mahmoodi

**Affiliations:** ^1^Assistant Professor, Department of Orthodontics, Faculty of Dentistry, Tabriz University of Medical Sciences, Tabriz, Iran; ^2^Assistant Professor, Department of Orthodontics, Faculty of Dentistry, Azad University, Tehran Branch, Tehran, Iran

**Keywords:** Active tieback, elastomeric, ligatures, orthodontics space closure, separator

## Abstract

***Background and aims.*** The purpose of this study was to evaluate initial force and force decay of commercially available elastomeric ligatures and elastomeric separators in active tieback state in a simulated oral environment.

***Materials and methods.*** A total of 288 elastomeric ligatures and elastomeric separators from three manufacturers (Dentaurum, RMO, 3M Unitek) were stretched to 100% and 150% of their original inner diameter. Force levels were measured initially and at 3-minute, 24-hour, and 1-, 2-, 3- and 4-week intervals. Data were analyzed by univariate analysis of variance and a post hoc Tukey test.

***Results.*** The means of initial forces of elastomeric ligatures and separators from three above-mentioned companies, when stretched to 100% of their inner diameters, were 199, 305 and 284 g, and 330, 416, 330 g; when they were stretched to 150% of their inner diameters the values were 286, 422 and 375 g, and 433, 540 and 504 g, respectively. In active tieback state, 11-18% of the initial force of the specimens was lost within the first 3 minutes and 29-63% of the force decay occurred in the first 24 hours; then force decay rate decreased. 62-81% of the initial force was lost in 4 weeks. Although force decay pattern was identical in all the products, the initial force and force decay of Dentaurum elastomeric products were less than the similar products of other companies (P<0.05). Under the same conditions, the force of elastomeric separators was greater than elastomeric ligatures of the same company.

***Conclusion.*** Regarding the force pattern of elastomeric ligatures and separators and optimal force for tooth movement, many of these products can be selected for applying orthodontic forces in active tieback state.

## Introduction

After introduction of elastomeric ligatures three decades ago to tie archwires to brackets, Bennet and McLaughlin proposed the use of elastomeric ligatures (modules) for moving tooth and closing space in active tieback or active ligature state.^1,2^ In passive tieback the ligature wire extends from the molar band to archwire hook but in active tieback, the ligature wire extends from the elastomeric ligature in the molar band hook to archwire hook for delivery of force for space closure.^3^ Easy use of active tieback ligature for delivery of force, low price, easy cleaning procedures for patients and finally proper function in most clinical situations have led to their widespread use.^3,4^ Regarding the structural similarities between elastomeric separators and elastomeric ligatures, it seems possible to use these elastomeric products in active tieback state, too.

Polyurethane is the main ingredient in elastomeric products (elastomeric ligatures, separators and chains) and water absorption facilitates sliding of molecules and polymeric chains in these products, leading to rapid force degradation.^1^ Not only a large number of studies have been carried out on the properties and mechanical behavior of elastomeric chains but also the role of factors such as time, temperature, saliva pH and water absorption have been evaluated.^5-8^

Taloumis et al^9^ evaluated seven different brands of elastomeric ligatures used to tie archwires to brackets and concluded that different products undergo different patterns of force decay. In that study the highest force decay (42-66%) was observed within the first 24 hours but force decay rate decreased after that. Force degradation of elastomeric chains are related to the length of the filament and the number of loops in polyurethane structure.^10^

Although proper initial force and anticipation of force decay rate with time are considered important in all the force application systems and space closing procedures to achieve proper tooth movement without injuries to the periodontium and root structure,^11^ the results of a study by Nattrass et al revealed that clinicians apply different amounts of force in identical situations depending on the force delivery system they use.^12^ Therefore, measurement and standardization of the forces applied in all the force application systems are of utmost importance. McLaughlin tried various force levels during space closure and reported that a range of 150-200 gm is most effective. This minimizes any tendency for unwanted bite deepening and allows for efficient sliding mechanics and space closure. Active tiebacks are used to deliver a force of this size.^3^

The purpose of the present study was to evaluate and compare the initial force applied and the pattern of force decay in two commonly used elastomeric products, including elastomeric ligatures and elastomeric separators, in active tieback state, when stretched to 100% and 150% of their initial inner diameter in a simulated oral environment.

## Materials and Methods

In the present study 288 elastomeric products, including 48 clear elastomeric ligatures and 48 blue elastomeric separators with three different brands were evaluated (Table 1).

**Table 1:** Elastomeric ligatures and elastomeric separators

**Product**	**Manufacturer**	**Brand name**	**Size**	**Color**
**Ligature**	3M Unitek	Alastic A_1_ module	1.1	Clear
	Dentaurum	Dentalastic plastic ligature	1.35	Clear
	RMO	O-ring ligature	1.4	Clear
				
**Separator**	3M Unitek	S module	2	Blue
	Dentaurum	Dentalastic separator	2.1	Blue
	RMO	Separator Stick	2.2	Blue

By stretching the elastomeric ligatures and separators and measuring their initial force and then comparing the results with the proper forces suggested for tooth movement (150-200 gm),^3^ the products were selectively stretched to 100% and 150% of their initial inner diameter.

The 288 selected specimens were divided into 12 groups according to product type, the manufacturer and the amount of stretching, in such a manner that 24 specimens from each brand were stretched to 100% of the initial inner diameter and 24 others from the same brand were stretched to 150% of their initial inner diameter.

**Figure 1.**
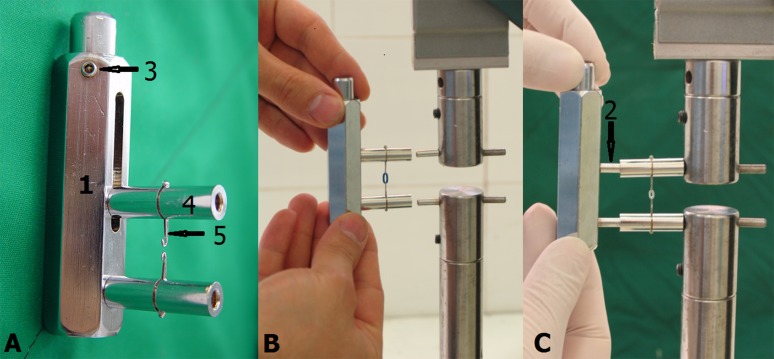
Holding appliance (A), main frame (1), long post (2), adjusting screw (3), tube (4) and steel hook (5). The placement of the tubes in the posts of holding appliance on one side (B). Specimen-containing tubes transferred to the holding appliance (C).

In order to hold the stretched specimens during the experiment, the holding appliance used by Nattrass et al^13^ was modified (Figure 1), which consisted of a main frame of chrome-plated brass measuring 70×18 cm. Two 12-mm-long posts were placed on this frame; one of the posts was fixed and the other one was movable. The distance between the two posts could be adjusted by a screw placed in the main frame. Two 28-mm-long chrome-plated brass tubes were fabricated to be placed on the posts; a steel hook was soldered to the middle of these tubes. These tubes and hooks made it possible to transfer the specimens to a universal testing machine (HSKS – 1417 Hounsfield Test Equipment LTD, England). It was possible to adjust the distance between the two facing hooks in each holding appliance by moving the movable post.

Attachments were devised and manufactured to be placed on the load cell and the lower fixed part of the universal testing machine, which consisted of two stainless steel cylindrical parts with a post, similar to posts in the holding appliance, at the end of each post. The distance between these posts was adjustable by the universal testing machine.

To stretch each specimen, the tubes in the holding appliance were placed on the attachment posts of the universal testing machine. Then, by moving the attachment on the load cell of the machine, the distance between the facing hooks was adjusted, using digital caliper (with 0.05 mm sensitivity/accuracy), to precisely correspond to the inner diameter of the specimen involved.

Subsequent to the placement of the specimen in the hooks, the machine stretched the specimens to 100% or 150% of their inner diameter at a strain rate of 0.2 inch/min to create a similar condition‏.

After recording the initial force, the specimens were kept in this stretched state for 3 minutes. Then the force was again recorded. After adjusting the distance between the posts in the holding appliance, corresponding to the distance between attachment posts on the universal testing machine (using digital caliper), these holding appliance posts were pushed into the tubes from the opposite direction to ensure the transfer of specimen-containing tubes to the holding appliance without any changes in stretch pattern (Figure 1).

The force of each specimen was measured after 24 hours and 1, 2, 3 and 4 weeks. The distance between the posts was separately measured for each group and the force was measured and recorded each time a specimen was transferred to the universal testing machine and transferred back to the holding appliance. All the holding appliances and specimens were kept in steel containers in artificial saliva (the formula used by Retief et al) at 37°C in the intervals between force measurements.^14^

One-sample Kolmogorov-Smirnov test was used to evaluate the normal distribution of data and univariate analysis of variance (UNIANOVA) was used to evaluate differences in the means of the forces and force decay percentages in different groups. Finally, a post hoc Tukey test was used to compare the groups.

## Results

The means of the initial and residual forces and force decay percentages of Dentaurum, RMO and 3M Unitek elastomeric ligatures and elastomeric separators stretched to 100% and 150% of their initial inner diameters at the experimental intervals are presented in Table 2. The means of initial forces of elastomeric ligatures and separators from the three above-mentioned companies, when stretched to 100% of their inner diameters were 199, 305 and 284 g and 330, 416 and 330 g, and when stretched to 150% of their inner diameters were 286, 422 and 375 g and 433, 540 and 504 g, respectively. In the specimens tested, nearly 11-18% of the initial force was degraded within the first 3 minutes and after 24 hours force degradation in elastomeric ligatures and elastomeric separators was 36-63% and 29-57%, respectively. This force decay after 24 hours resulted in a decrease in differences in the means of residual forces in different brands of elastomeric ligatures, because RMO in ligature and 3M in separator products had produced higher forces and underwent higher force degradation compared to others; the means were 118-158 g and 137-222 g in ligatures stretched to 100% and 150% of their inner diameters, respectively. The resisual forces in elastomeric separators stretched to 100% and 150% of their inner diameter were 156-234 g and 217-270 g after 24 hours, respectively. After 24 hours, force decay rate decreased in all the specimens.

**Table 2:** Means of the initial force and force decay percentages of elastomeric ligatures and separators stretched to 100% and 150% of their inner diameter

		**Extension** **%**	**0 hours** **Mean (SD)**	**3 min** **Mean (SD)**	**24 hours** **Mean (SD)**	**1 week** **Mean (SD)**	**2 weeks** **Mean (SD)**	**3 weeks** **Mean (SD)**	**4 weeks** **Mean (SD)**
**Ligatures**	Dentaurum	100	199.5 (20.5)	174.5 (18.8)	127.5 (26.3)	107.0 (22.5)	88.7 (19.3)	83.7 (17.5)	79.5 (18.4)
	%	100	—	12.5 (2.3)	36.6 (8.5)	46.6 (8.3)	55.7 (7.4)	58.2 (6.3)	60.4 (6.7)
	Dentaurum	150	286.6(15.5)	242.9 (9.0)	174.5 (19.1)	145.4 (16.7)	125.8 (16.2)	118.7 (13.3)	110.4 (11.1)
	%	150	—	15.1 (1.7)	39.1 (5.7)	49.3 (4.4)	56.1 (4.5)	58.5 (3.8)	61.4 (3.4)
	RMO	100	305.0 (23.7)	271.6 (25.1)	157.9 (17.7)	138.3 (18.5)	120.0 (21.3)	112.0 (23.3)	107.5 (21.6)
	%	100	—	10.9 (2.7)	48.1 (4.9)	54.6 (5.2)	60.6 (6.4)	63.2 (6.7)	64.8 (6.1)
	RMO	150	422.9 (16.9)	364.1 (19.6)	222.5 (20.1)	193.3 (27.1)	170.8 (20.8)	152.5 (20.6)	144.5 (18.7)
	%	150	—	13.9 (1.6)	47.3 (4.9)	54.2 (6.5)	59.5 (5.1)	63.8 (4.9)	65.7 (4.5)
	3M Unitek	100	284.5 (16.8)	247.0 (14.3)	118.7 (30.8)	80.4 (21.1)	65.4 (20.1)	61.2 (20.7)	57.5 (20.8)
	%	100	—	13.1 (1.7)	58.4 (9.7)	71.8 (6.8)	77.0 (6.6)	78.5 (6.7)	79.9 (6.8)
	3M Unitek	150	375.8 (16.3)	317.5 (18.2)	137.9 (10.5)	94.5 (11.1)	76.6 (5.7)	70.0 (8.2)	68.3 (8.3)
	%	150	—	15.5 (2.4)	63.2 (2.6)	74.8 (2.9)	79.5 (1.6)	81.3 (2.1)	81.7 (2.3)
									
**Seprator**	Dentaurum	100	330.0 (13.9)	275.8 (14.5)	234.1 (16.7)	173.3 (16.8)	163.3 (15.7)	157.0 (16.4)	149.5 (17.1)
	%	100	—	16.4 (2.09)	29.0 (4.1)	47.5 (4.1)	50.5 (3.6)	52.4 (4.3)	54.6 (4.6)
	Dentaurum	150	433.7 (15.5)	360.8 (23.4)	270.8 (23.7)	235.8 (24.0)	203.3 (21.1)	192.0 (16.4)	184.5 (15.4)
	%	150	—	16.8 (1.7)	37.4 (5.7)	45.5 (5.2)	53.1 (4.4)	55.6 (3.3)	57.4 (3.0)
	RMO	100	416.2 (12.8)	350.0 (10.4)	222.0 (12.6)	169.5 (14.5)	142.9 (12.8)	135.4 (13.7)	131.2 (11.8)
	%	100	—	15.9 (1.4)	46.6 (2.9)	59.2 (2.8)	65.6 (2.6)	67.4 (3.0)	68.4 (2.6)
	RMO	150	540‏.0 (16.2)	443.7 (18.1)	247.0 (18.0)	207.0 (21.5)	170.0 (25.6)	153.7 (22.4)	144.1 (15.3)
	%	150	—	17.8 (1.3)	54.2 (2.7)	61.6 (3.2)	68.5 (4.1)	71.5 (3.7)	73.3 (2.5)
	3M Unitek	100	330.8 (11.8)	282.0 (13.0)	156.2 (22.7)	121.2 (20.5)	110.8 (22.3)	100.0 (17.7)	96.2 (16.9)
	%	100	—	14.7 (2.1)	52.7 (6.5)	63.3 (6.1)	66.4 (6.6)	69.7 (5.2)	70.9 (4.9)
	3M Unitek	150	504.5 (25.2)	409.1 (20.2)	217.0 (26.4)	161.2 (26.8)	147.9 (29.5)	136.6 (27.4)	126.2 (25.8)
	%	150	—	18.8 (1.3)	56.9 (4.7)	68.0 (4.8)	70.7 (5.4)	72.9 (4.9)	74.9 (4.7)

**Figure 2.**
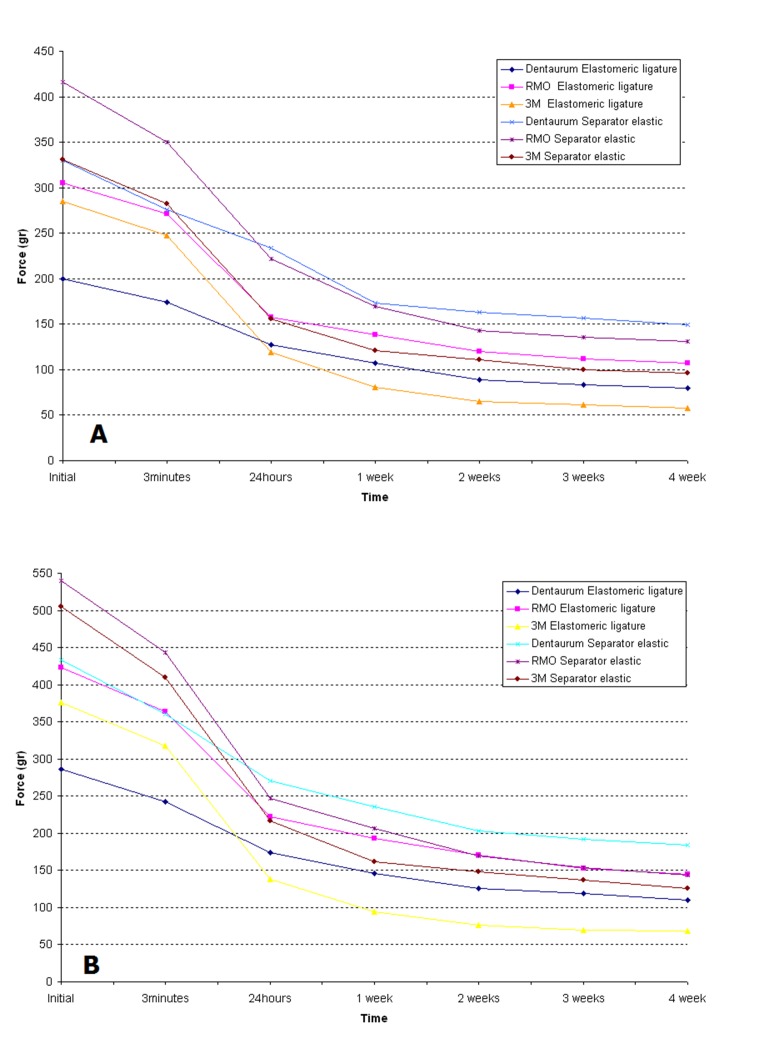
Means of the initial and residual forces in elastomeric ligatures and elastic separators stretched to 100% (A) and 150% (B) of their inner diameter.

After 4 weeks, 60-81% of the initial force in elastomeric ligatures and 54-75% of the initial force in elastomeric separators had decayed, resulting in a resisual force of 57-107 g and 68-114 g in elastomeric ligatures stretched to 100% and 150% of their inner diameter, respectively, and 96-149 g and 126-184 g in elastomeric separators stretched to 100% and 150% of their inner diameter, respectively. After 28 days none of the specimens completely lost its force (Figure 2).

**Figure 3.**
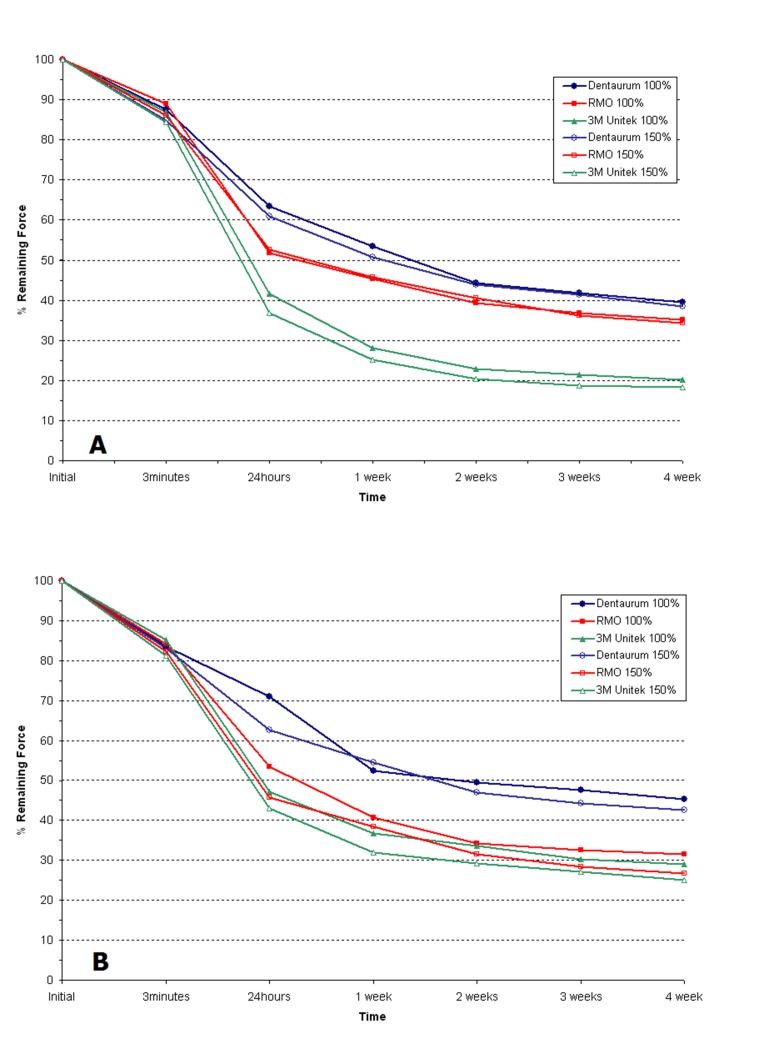
Means of residual forces in elastomeric ligatures (A) and elastomeric separators (B) stretched to 100% and 150% of their inner diameter.

Force degradation pattern of elastomeric ligatures and elastomeric separators manufactured by the three above-mentioned companies is shown in Figure 3.

3M Unitek elastomeric ligatures and elastomeric separators lost more force in 4 weeks, compared to similar products of RMO and Dentaurum (P < 0.05).

Except for the first 3 minutes, in all the intervals Dentaurum elastomeric ligatures and elastomeric separators demonstrated less force degradation compared to similar products of the two other companies (P < 0.05). Under 100% and 150% stretch patterns, the initial forces of elastomeric separators of one brand were greater than those of elastomeric ligatures of the same brand but force decay patterns were almost similar at all the intervals. The role of different and independent contributing factors (the manufacturer, product type, time and the extent of stretching) in force of elastomeric products, using Univariate ANOVA are summarized in Table 3. Statistical analysis showed significant differences with regard to the companies (P < 0.001), product type (ligature or separator), time interval and amount of stretching (P < 0.001). In addition, interaction effects of variables was statistically significant (P < 0.001).

**Table 3:** The role of independent variables (the manufacturer, product type, time and the extent of stretching) in the amount of force exerted by elastomeric ligatures and elastic separators using univariate ANOVA

	**Type III Sum of Squares**	**df**	**Mean Square**	**F**	**Sig.**
**Corrected Model**	24184248.165(a)	83	291376.484	823.407	.000
**Intercept**	80092931.002	1	80092931.002	226336.323	.000
**com**	869804.861	2	434902.431	1229.000	.000
**product**	2135254.018	1	2135254.018	6034.060	.000
**extent**	1471716.716	1	1471716.716	4158.956	.000
**Time**	17554194.345	6	2925699.058	8267.795	.000
com ^*^ product	221250.298	2	110625.149	312.618	.000
com ^*^ extension	2117.956	2	1058.978	2.993	.050
product ^*^ extent	13054.018	1	13054.018	36.890	.000
com ^*^ product ^*^ extent	68803.869	2	34401.935	97.217	.000
com ^*^ Time	910043.750	12	75836.979	214.309	.000
product ^*^ Time	285807.440	6	47634.573	134.612	.000
com ^*^ product ^*^ Time	61895.536	12	5157.961	14.576	.000
extent ^*^ Time	521526.687	6	86921.114	245.632	.000
com ^*^ extent ^*^ Time	36316.766	12	3026.397	8.552	.000
product ^*^ extent^ * ^Time	22492.163	6	3748.694	10.594	.000
com ^*^ product ^*^ extent ^*^ Time	9969.742	12	830.812	2.348	.005
**Error**	683670.833	1932	353.867		
**Total**	104960850.000	2016			
**Corrected Total**	24867918.998	2015			

## Discussion

The initial forces produced by elastomeric ligatures and elastomeric separators, manufactured by Dentaurum, RMO and 3M Unitek, in active tieback state when stretched to 100% and 150% of their initial inner diameter demonstrated statistically significant differences in most cases. These differences might be attributed to differences in structural components, physical dimensions of the products, and differences in the amount of stretching due to minor differences in the inner diameter of the different products.

In all the specimens, 11-18% of the initial force was lost within the first 3 minutes, but there were no statistically significant differences between the specimens. Considering the fact that in previous studies the amount of force decay in such a short time has almost been ignored, this force decay requires more attention to evaluate the initial force in clinical circumstances, so that appropriate forces can be employed in such situations, considering this amount of force degradation.

In the present study, the means of force decay for 3 different brands of elastomeric ligatures in the first 24 hours were approximately 36% (Dentaurum) to 63% (3M Unitek), which is almost similar to previous studies on elastomeric chains.^5-8^

Force decay pattern of 3M Unitek ligatures in our study was almost similar to the results of a study by Taloumis et al.^9^ In that study elastomeric ligatures were used to tie archwires to brackets, but our study simulated tieback force delivery system. In both studies in all the intervals force decay of 3M Unitek elastomeric ligatures was more than the products of other companies, consistent with the results of other studies on force decay of Alastic elastomeric chains manufactured by 3M Unitek.^15,16^

In a period of 4 weeks, force decay of RMO elastomeric ligatures in our study was less than that in the study carried out by Taloumis et al^9^ but it was more than the force decay of RMO chains in the studies carried out by De Genova et al^8^ and Lu et al.^17^ Some of the differences between the results of the present study and a study carried out by Taloumis et al^9^ might be attributed to differences in the methods used in these studies.

The initial force of none of the elastomeric ligatures evaluated completely disappeared after 28 days, which is consistent with the results of a similar study by Samuels et al.^18^ Since different amounts of force are required in different clinical situations, it is possible that in some cases the amount of residual force after 28 days is still sufficient for tooth movement, although the influence of oral environment and tooth movement on the amount at remaining force should be taken into account. There were significant differences in tensile properties of different brands of ligatures. The decrease in strength properties of elastomeric ligatures shows that they should be replaced at each appointment to reduce the risk of rupture.^19^

Despite the fact that the amount of initial force in Dentaurum elastomeric separators and elastomeric ligatures was less than that in the products manufactured by two other companies, both Dentaurum products underwent less force decay compared to RMO and Unitek products, indicating milder forces with less force decay in the products of Dentaurum Company.

Minor differences during a 4-weeks period in the force decay patterns of elastomeric separators and elastomeric ligatures manufactured by one company indicate structural similarities of elastomeric ligatures and elastomeric separators of that company.

However, in some cases the initial forces of elastomeric ligatures and elastomeric separators stretched to 100% and 150% of their inner diameter may seem too great for clinical situations.^20^ Tieback method of space closure has more appropriate initial force and slower force decay than elastomeric chains.^21^ According to Figure 2 and McLaughlin suggestion for optimal force in active tieback, some of these products seem to be appropriate in active tieback state for the purpose of applying force to move teeth considering relatively significant force decay in the first 24 hours and mild and continuous force decay within 4 weeks after their application. Brown evaluated in vitro force decay of three elastomers in active tieback state and showed 63-83% force decay after four weeks. Force decay pattern of our study is similar to his study.^22^

The results of the present study provide relevant information regarding the amount of force applied by this system in clinical situations and the amount of force decay in different intervals, so that clinicians can apply proper amounts of force in different clinical situations by gaining knowledge on the forces produced by different products under different stretch patterns.

## Conclusion

Considering the initial force and force decay patterns in elastomeric ligatures and elastomeric separators, approximately 11-18% of the initial force was lost within the first 3 minutes, and 29-63% of force decay occurred within the first 24 hours. After four weeks 19-48% of the initial force was still remaining.

## Acknowledgements

The authors would like to extend their appreciation to the Office of the Vice Chancellor for Research, Tabriz University of Medical Science for financial support of this study.
